# Analysis of aroma compounds in barbecued mutton during storage and exploration of customer preferences

**DOI:** 10.1186/s13065-026-01851-9

**Published:** 2026-05-29

**Authors:** Aygul Alim

**Affiliations:** School of Life Science, Zhuhai College of Science and Technology, Jinwan, Zhuhai China

**Keywords:** Kebab, Storage period, GC-IMS, Aroma compounds, Molecular docking

## Abstract

Kebab (barbecued mutton) is a traditional meat product with a rich roasted meaty aroma and is highly preferred by consumers. In this study, the volatile flavor profiles of Kebab (barbecued mutton) were analyzed employing HS-GC-IMS and sensory evaluation techniques. Simultaneously, molecular docking was used to explore the mechanism of six key aroma compounds which are important for consumer pleasure and to infer and enhance the optimal storage conditions. A total of 61 volatile compounds were identified by HS-GC-IMS. The PCA demonstrated a correlation between the storage time and the volatile compounds in different tissue such as Lean (KL), fat (KF) and mixed (KM) of Kebab. The main characteristic compounds in sample KL are 2-hexanal, 2-nonanal, E-2-pentanal, 2-pentylfuran, butyl acetate, butyl 2-methylbutyrate, and 2-heptanone. Sample KF is characterized by trimethylamine, triethylamine and 1-hydroxy-2-propanone as its main compounds, while sample KM contains 2-octanol, 2-methylbutanal, and 2,3-butanedione as its main ones. When the storage time reached 28 days, sample KL maintained strong meaty and nutty aromas; in samples KM and KF, the fatty, sulfur, and sour aromas increased significantly. Compounds such as 5-methylfurfural, 2-pentylfuran, (E)-2-hexenal, 2-octanone, trans-2-nonanal, and 3-methylbutanol can cross the blood-brain barrier, thereby inducing a sense of pleasure. It is recommended that lean meat should be used in the processing of roasted meat products, as this helps maintain a pleasant meat flavor and reduces off-odors. The research offers theoretical foundation for the development of Kebab products.

**Graphical abstract:**

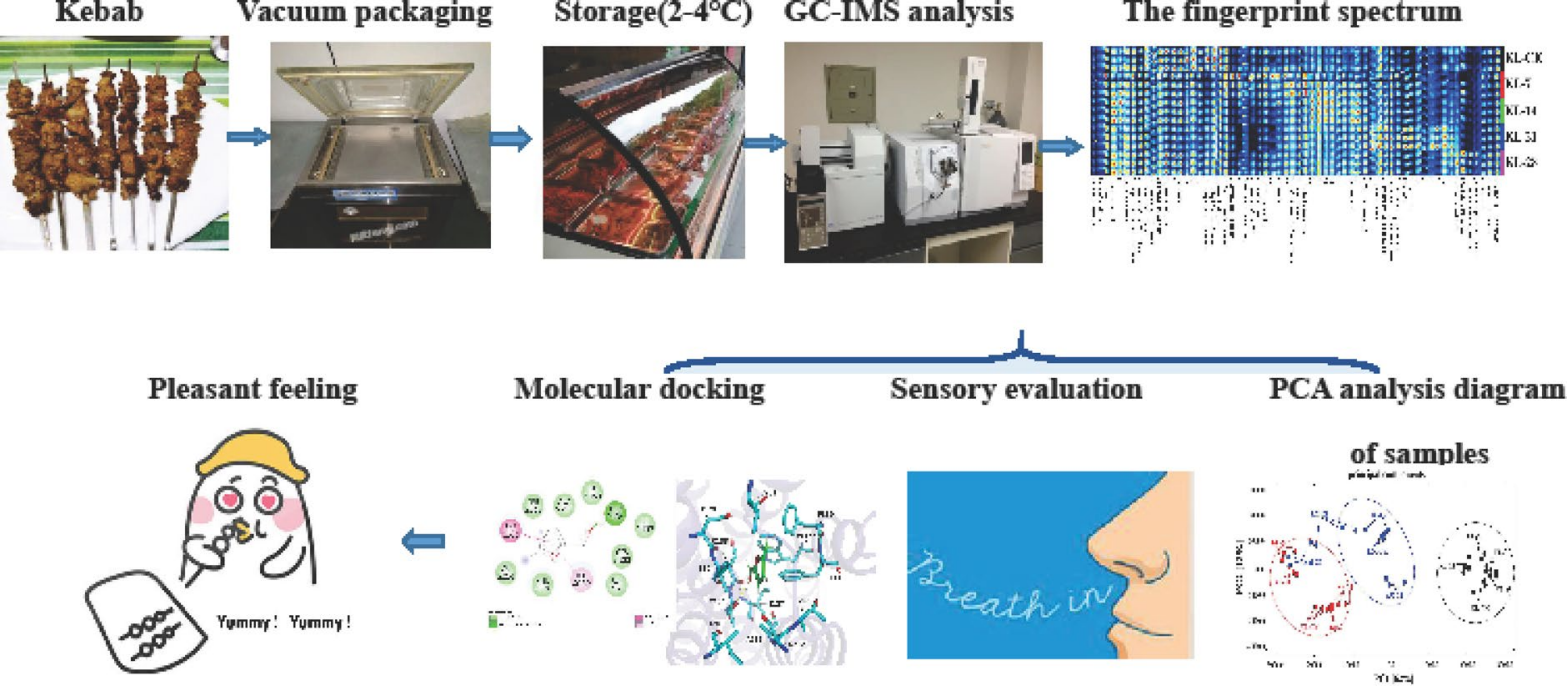

## Highlights

1. GC-IMS analyzed dynamic changes in aroma profiles of three kebab types (lean, fat-lean, fatty) throughout storage.
2. The PCA demonstrated a correlation between the storage time and the volatile compounds.
3. Molecular simulations elucidated the hedonic mechanism of key meaty aroma compounds.

## Introduction

As a traditional meat product of ethnic groups such as the Uyghur people, Kebab (barbecued mutton) has unique characteristics in color, aroma, taste, and shape. Consumers often evaluate product quality on the basis of above mentioned characteristics. The level of lipid oxidation in meat is closely linked to its edibility and storage characteristics and is also a major prerequisite for the formation of meat flavor [1]. Kebab is a nutritious and healthy food with high protein, low fat, and low cholesterol contents. It is equally easy to make it, delicious, and affordable. Kebab can be used as a street-style fast food or a delicious dish for entertaining guests. It meets the life requirements of people in this modern age. Given the enormous consumer market demand, relevant research on barbecued mutton is extremely required. However, owing to the scarcity of packaged barbecued mutton products and the lack of unified relevant parameters, it is difficult to carry out innovative industrial production.

GC-IMS is an emerging gas separation and detection technology that combines the high separation capacity of gas chromatography with the rapid response feature of ion mobility spectrometry (IMS). This combination endows GC-IMS with rapid analysis, high sensitivity, and variable-volume injection ability, all without the need for pretreatment [2]. In recent years, GC-IMS has developed rapidly in food flavor analysis and has been used in various food research fields, including establishing fingerprint spectra of volatile flavor compounds for food classification and adulteration detection [3, 4], investigating the changes in volatile substances during meat processing and volatile components during storage [5–8], evaluating the freshness and degree of spoilage of food [9], and detecting food odors [10, 11]. In the research on the flavor of grilled lamb shashliks, Yao [12] applied HS-SPME-GC-MS, SPME-GC×GC-TOF-MS, HS-GC-IMS, E-nose, and electronic tongue systems in a feasibility study on flavor characterization of five commercial Chinese grilled lamb shashliks. Additionally, Shen [13] analyzed the volatile flavor profiles of grilled lamb seasoned with salt, chili pepper, and cumin employing HS-SPME-GC-MS, HS-GC-IMS, E-nose, and sensory evaluation techniques. However, GC-IMS technology has not been used to characterize the fingerprint of volatile flavor substances in different tissues (lean meat, fat meat, and fat-lean mixed meat) of Kebab during storage.

Regarding previous research on mutton flavor, studies on lamb shashliks have shown that a total of 67 volatile compounds were identified via HS-SPME-GC-MS, and 59 via HS-GC-IMS. PCA analysis revealed a correlation between seasonings and volatile compounds, with five principal components accounting for 99.54% of the total variance [12]. In addition, Shen [13] identified 198 volatile organic compounds (VOCs) using data fusion techniques. They also developed five predictive models to analyze the VOC composition and achieve brand identification of lamb shashliks. GC-MS was employed to analyze the volatile components of three adipose tissues, perirenal fat (PRF), caudal subcutaneous fat (CSCF), and heart fat (HF), harvested from 16 lambs, the resulting data identified 130 volatile compounds, which are capable of distinguishing between the two dietary types in one or more of the three tissues [14]. Pandy [15] investigated the effects of processing conditions, including processing time, temperature, and cooking methods on the physicochemical and textural properties of shami kebab. Gulen [16] investigated the changes in lipid fraction induced by thermal treatment during the production of Doner kebab.

In recent years, computer-based molecular modeling techniques such as molecular docking and molecular dynamics have received widespread attention because of their low cost and high time efficiency. Wang [17] used molecular docking to study the antidepressant mechanism of fluoroscopolamine analogs. Xia [18] constructed an “REO active ingredient-key target-depression” interaction network by predicting the active aroma compounds of rose essential oil (REO) and depression-related targets, further revealing the regulatory mechanism of REO in treating depression. Chandharakoo [19] evaluated the effect of citrus essential oil on sleep activity by electroencephalography (EEG) and then studied sleep onset latency to confirm its effect on sleep activity.In the context of odor-induced taste enhancement (OITE) and sugar reduction, these techniques have been used to explore odorants, and it has been indicated that beef odors potentially lead to a sense of pleasure [20].

To date, some studies have reported the key aroma compounds in Kebab, but the flavor differences among different mutton Kebab tissues have not yet been reported as well as the underlying molecular mechanisms by which they induce a sense of pleasure, have not yet been fully elucidated. In this study, GC-IMS technology was employed to detect the aroma compounds in barbecued mutton (kebab) samples. The detection was conducted once a week during low-temperature refrigeration, spanning a total of 28 days, with 5 tests performed for volatile compounds. On this basis, comparisons were made regarding changes in aroma compounds among lean meat, fat meat, and fat-lean mixed meat. Principal component analysis (PCA) was employed to analyze the correlations between different sample groups. Furthermore, sensory evaluation was conducted to assess flavor changes across various storage periods. These investigations provide a theoretical foundation for studying flavor dynamics in processed mutton products and extending the storage life of kebab, while also laying groundwork for exploring optimal mutton raw material selection to preserve desirable flavor in mutton products during storage. Additionally, six key aroma compounds: 5-methylfurfural, 2-pentylfuran, (E)-2-hexenal, 2-octanone, trans-2-nonanal, and 3-methylbutanol, were chosen for further analysis. Through the application of molecular docking simulation technology to explore why consumers favor barbecued meat, and a preliminary investigation was conducted into the physiological mechanisms by which eating barbecued meat enhances mood and induces pleasure.

## Materials and methods

### Materials and instruments

Sheep were grazed and raised in Altay City, from which male sheep were randomly selected. These sheep were slaughtered uniformly based on the criteria of age (18–30 months), health status and body weight (> 16 kg). After slaughter, the carcasses were randomly allocated and transported to Urumqi within 12 h under low-temperature conditions (frozen at − 20 °C). Subsequently, the leg meat from both left and right sides was removed from these carcasses and stored at the same low temperature in Urumqi Friendship Supermarket. The barbecue (kebab) restaurant (Urumqi, China.) purchased fresh meat from the supermarket to make barbecued meat for this study. The lab instruments for the analysis were a Flavor Spec ^®^ gas phase ion mobility spectrometer manufactured by G.A.S. Gesellschaft für analytische Sensorsysteme mbH, Germany.

## Methods

*Sample preparation for experiments:* The ordinary method of roasting mutton involves randomly selecting deboned mutton, mixing fat and lean meat, adding salt and spices (crystalline powder, chili powder) and then roasting. Therefore, the roasted mutton in this experiment was divided into lean meat only, fat meat only, and fat-lean mixed meat (KL, KF, and KM), respectively. A total of 500 g of samples were collected and divided into the three aforementioned types. Given that the storage period of the barbecued meat is 28 days, sampling was conducted once weekly for a total of five times, with three parallel samples prepared each time and 4.5 g (accurate to 0.01 g) of each parallel sample was weighed and then vacuum-packed in aluminum foil bags and stored at low temperature (0–4 °C) for later use. Samples were taken once a week. Each sample having weight of 3 g were accurately weighed, placed in a 20 mL headspace vial, and sealed HS-SPME-GC–MS analysisn.

*HS-SPME-GC-IMS determination*: The test conditions were adapted from reference with minor modifications [13]. The analysis utilized a column of type FS-SE-54-CB-1 (15 m × 0.53 mm, 1 µM), the column temperature was 60 °C; automatic headspace injection: the injection volume was 500 µL. The samples were incubated for 20 min at a temperature of 65 °C, with a rotation speed set at 500 r/min. The needle temperature for injection was maintained at 85 °C. The carrier gas was nitrogen, with an analysis time of 30 min.The flow rate was initially 2 mL/min and was maintained for 2 min; then, the flow rate linearly increased to 15 mL/min within 10 min, increased to 100 mL/min within 20 min, and then increased to 150 mL/min within 30 min. Then, the data processing began.

### Sensory evaluation

Sensory evaluation was performed in three different sessions inside a sensory evaluation room at 20–24 °C. The sensory evaluation team had 12 trained panellists (aged 23–40 years; 50% female and 50% male; 5 teachers and 7 graduate students at our College of Life Sciences; this demographic information was added as a covariate in the statistical analysis). On the evaluation day, the samples for all the sessions were placed under the same conditions. A total of 12 panelists were present in each session, with 3 rounds of testing. All the panelists had two weeks of sensory evaluation training. According to relevant references [13, 21], the characteristic indicators for sensory evaluation are selected as the following six: meaty, nutty, fatty, green, sulfur and sour. The characteristics of the samples were scored via a 10-point system. The intensities of the aroma was ranked on a 10-point scale (0–2, very weak; 3–4, weak; 5–6, medium; 7–8, strong; 9–10, very strong [15]. The experiments were performed in triplicate, and the average scores of each trait were used for the statistical analysis.

### Molecular docking for preliminary exploration of pleasure induced by aroma compounds in kebab

The crystal structure of the serotonin receptor (PDB ID: 6G79) was obtained from the RCSB PDB. The SDF structures of 6 characteristic aroma compounds from Kebab were obtained from the PubChem database. The MMFF94s force field is subsequently used for geometric optimization to eliminate unreasonable bond lengths and bond angles. Semiflexible molecular docking was carried out via the AutoDock Vina program [22] and the cocrystallized small-molecule agonist binding region was used as the conformational search space (center_x = 94.3, center_y = 52.2, center_z = 64.3) to explore potential geometric sites. Subsequently, semiflexible molecular docking was performed using the Vina procedure. After docking, interaction analysis was performed using the PyMOL program.

### Statistical analyses

For this study, three independent batches of the grilled lamb samples were prepared, with each batch being measured in triplicate. Software tools including the Laboratory Analytical Viewer (LAV), two plugins (Reporter and Gallery Plot), and the GC-IMS Library Search tool. The statistics and analysis of the data were conducted via Office Excel 2007. The multimodal model Deep-B3 tool was used to predict flavor compounds.

## Results

### Comparison of flavor compounds in different samples via GC-IMS

**Fig. 1:**
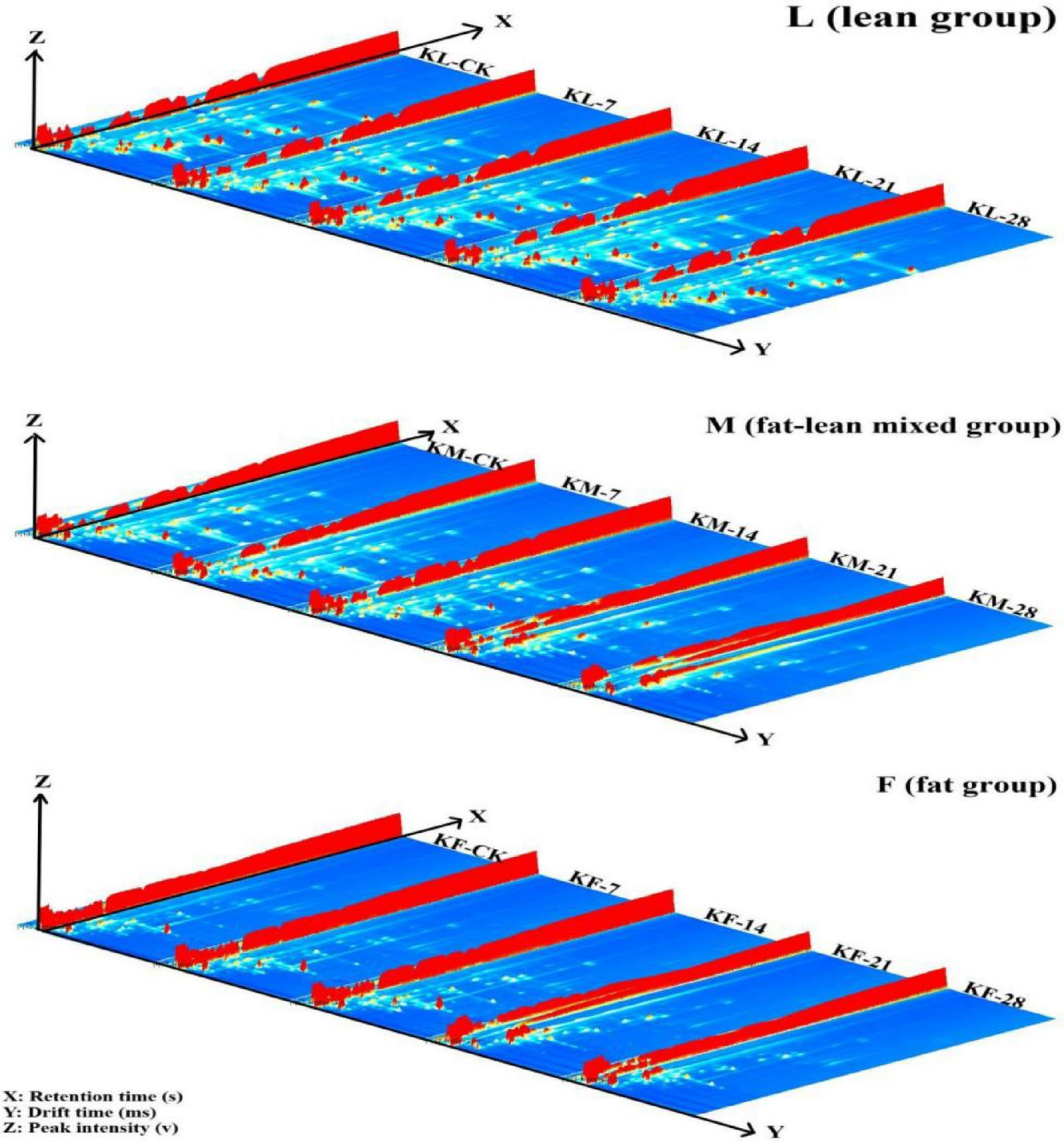
GC-IMS three-dimensional map of different tissues of Kebab samples during storage

The Reporter plugin of GC-IMS was used to generate GC-IMS three-dimensional maps of volatile compounds in different tissues of Kebab (barbecued mutton) samples during storage. The three-dimensional map of the GC-IMS data shown in Fig. 1 includes the retention time, transfer time, and peak intensity. In Fig. 1, each coordinate represents a different retention time, migration time, and peak intensity, and each peak signal represents a volatile organic compound, while the three-dimensional image displays the presence of volatile organic compounds. As shown in Fig. 1, by measuring the residual time and migration time in a sample, different volatile components can be identified, and peak intensities can be compared between different compounds. Two-dimensional GC-IMS spectra are more simple and easier to read, providing an accurate and comprehensive image of the characteristics and intensity of aromatic compounds, which is very useful for in-depth statistical analysis.

**Fig. 2:**
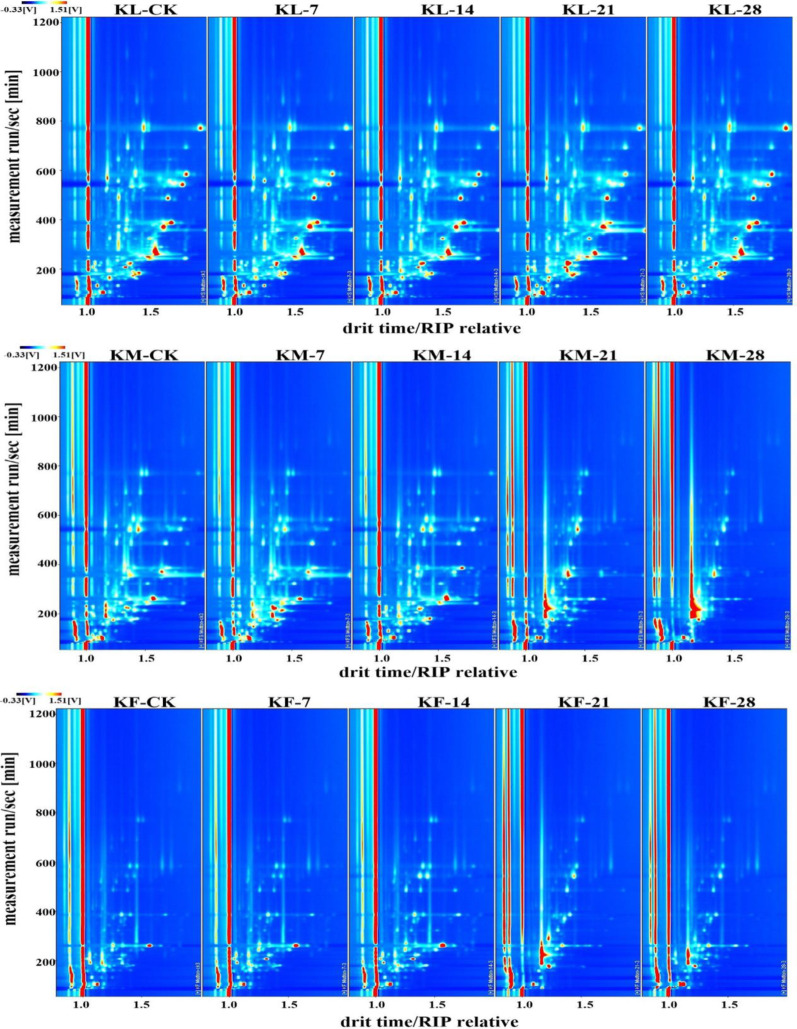
Comparison of HS-GC-IMS two-dimensional spectra of different tissues of Kebab samples during storage (vertical view)

Figure 2 shows the GC-IMS spectra (vertical view) of different tissues from the Kebab samples at different storage times, including KL-CK (storage day 0), KL-7, KF-7, KM (storage day 7), KL-14 (storage day 14), KL-21 (storage day 21), and KL-28 (storage day 28). The background of the entire graph is blue, with a horizontal axis of 1.0. The red vertical axis represents the RIP peak (the standardized reaction ion peak), the vertical axis represents the gas chromatography retention time, and the horizontal axis represents the ion migration time (standardized process). There are many points on both sides of the RIP peak, with one point for each volatile compound [23, 24]. The colors of the two dots on both sides of the RIP peak represent the concentrations of red dots and light blue substances in the volatile organic roasted lamb sample. White indicates a lower substance content, red indicates a higher substance content, and darker peak colors indicate higher substance concentrations. Figure 2 shows that the number and area of color spots on days 0 and 28 are significantly greater than those on days 7, 14, and 21 of storage, indicating a significant increase in the content of volatile substances on days 0 and 28. As shown in Fig. 2, there were significant differences in the volatile substance content of each sample. The red color of different tissues in lamb on day 0 was clearly greater than that on days 7, 14, 21, and 28, indicating that roasted lamb (stored on day 0) produced a large amount of volatile compounds. To clearly compare different samples, the following are the fingerprint spectra of each peak [25].

**Fig. 3:**
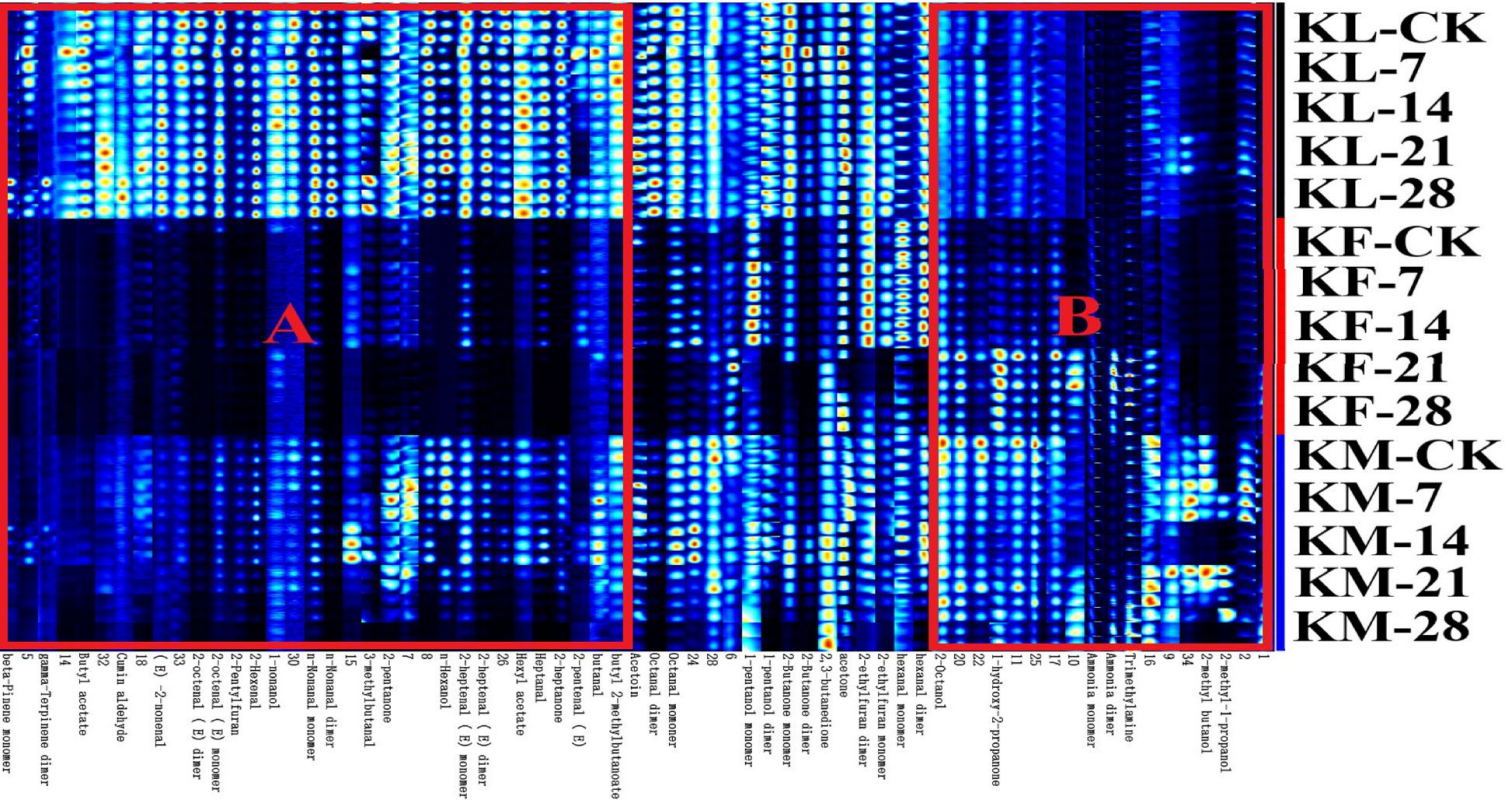
Gallery plot (fingerprint map)

As shown in Fig. 3, through the fingerprint spectrum, the commonalities and differences in the composition and content of flavor substances among the three groups of kebab tissues during the storage period can be visually compared. In Fig. 3, each row represents the composition of volatile components detected in one sample, and each column represents the signal peak situation of the same volatile flavor component in different samples. The fingerprint spectrum is a comparison of volatile substances in three types of mutton during five stages within 28 days of storage. The three groups of samples and 15 different samples can be distinguished by volatile substances. Moreover, most of the volatile substances in lean meat (KL) have relatively high concentrations, and the concentrations of volatile substances during the entire storage period are higher than those in fat meat (KF) and the mixture of lean and fat meat (KM). A separate discussion of each group revealed that the volatile substances in each group of samples varied with different storage periods. In the KF group, KF-CK to KF-14 formed one group, and KF-21 to KF-28 formed another group. Among the three groups, the KF group clearly contained the fewest types of volatile substances. In the figure, the substances in area A have the highest concentration in KL, followed by those in KM, and the lowest or no substances are present in KF. The pattern of substances in this area may be related to the content of lean meat (KL). The substances in this area from left to right mainly include β-pinene, γ-terpinene, butyric acid acetate, cuminaldehyde, E-2-nonanal, E-2-octanal, 2-pentylfuran, 2-hexanal, nonanol, nonanal, 3-methylbutanal, 2-pentanone, hexanol, E-2-heptanal, hexyl acetate, heptanal, E-2-pentanal, butanal, butyl 2-methylbutyrate, acetoin, and octanal. In the figure, the substances in area B have relatively high concentrations during the storage period in KM, relatively high concentrations in the later storage period of KF, and relatively low or none during the entire storage period of KL. They mainly include 2-octanol, 1-hydroxy-2-propanone, ammonia, trimethylamine, triethylamine 2-methylbutanal, and 2-methyl-1-propanol. In the KM group, the volatile substances of the KM-14 sample clearly do not conform to the overall pattern. These results indicate that the volatile components of different tissues of roasted meat are different and that the types and contents of volatile components also change with storage time [25].

The fingerprint spectrum is a comparison of the volatile substances of the mutton in the KL group. There were significant differences in the quantities and concentrations of the volatile compounds among the five samples. The figure shows that there are different compounds in different storage periods; that is, some substances have the highest concentrations in specific stages and lower concentrations or are absent in other stages. For example, the aroma compounds in area A in the figure have the highest concentration in KL-CK. The compounds with the highest concentrations in KL-7 mainly include 2-butanone, 5-methyl-2-furancarbaldehyde, E-2-pentanal, 2-pentylfuran, butyl acetate, butyl 2-methylbutyrate, 2-heptanone, 2,3-butanedione, etc. The substances with relatively high concentrations in KL-14 mainly include nonanol, etc. The substances with the highest concentrations in KL-21 mainly include (E)2-nonanal, 2-pentanone, 1-hydroxy-2-propanone, (E)2-octanal and hexanol, etc. The substances with the highest concentrations in KL-28 mainly include 3-methylbutanal, β-pinene, γ-terpinene, 2-hexanal and 2-nonanal, etc. Because lean meat contains a relatively high amount of flavor precursors, it develops a rich flavor after roasting, thus maintaining a good meaty aroma during storage.

In the KF group, KF-CK to KF-14 formed one group, and KF-21 to KF-28 formed another group. The concentration in KF-CK was low, and the concentrations in KF-7 and KF-14 were the same and greater than those in KF-21 and KF-28. The volatile substances from left to right mainly include 2-butanone, 3-methylbutanal, 2-pentanone, 2-pentylfuran, octanal, hexyl acetate, nonanal, (E)2-octanal, heptanal, 2-ethylfuran dimer, pentanol, hexanal, ethyl hexanoate, (E)2-heptanal, (E)2-pentanal, and 2-hexanal, etc. The concentrations of the following compounds are the same in KF-21 and KF-28 and higher than those in the other samples, mainly trimethylamine, butyl 2-methylbutyrate, hydroxy-2-propanone, and ammonia, etc.

In the KM group, it is obvious that the KM-14 sample has a relatively large difference from the others. The compound concentrations were relatively high in KM-7 and KM-21. From left to right, the main compounds were 2-methylbutanal, 2-pentanone, 2-hexanal, acetoin, butanal, octanal, pentanol, acetone, 2-butanone, 3-methylbutanal, butyl acetate, heptanal, (E)2-pentanal, β-pinene, nonanal, and hexyl acetate, etc. The concentration was the highest in KM-28, with 1-hydroxy-2-propanone, 2,3-butanedione, 2-methyl-1-propanol, trimethylamine, ammonia, etc., being the main substances.

In terms of detecting the key aroma compounds of roasted mutton, 33 and 30 compounds were detected in mutton before and after roasting, respectively [9]. 3-Methylbutanal, pentanal, hexanal, heptanal, octanal, nonanal, and 1-octen-3-ol are important flavor compounds. Among them, hexanal and 1-octen-3-ol are key flavor compounds [26]. These results are similar to those of the present study, but there are no reports on the flavor changes of different tissues during the storage period. In conclusion, different kebab tissues all contain representative compounds, and the content of aroma components plays a dominant role during storage. Specifically, the main characteristic compounds in sample KL include 2-hexanal, 2-nonanal, E-2-pentanal, 2-pentylfuran, butyl acetate, butyl 2-methylbutyrate, and 2-heptanone. The KF was characterized by pentanol, 2-ethylfuran dimer, hexanal, and hydroxy-2-propanone as its main compounds, in sample KM were 2-octanol, 2-methylbutanal, and 2,3-butanedione.

### Qualitative analysis of aroma compounds in samples (GC×IMS library search)

**Fig. 4:**
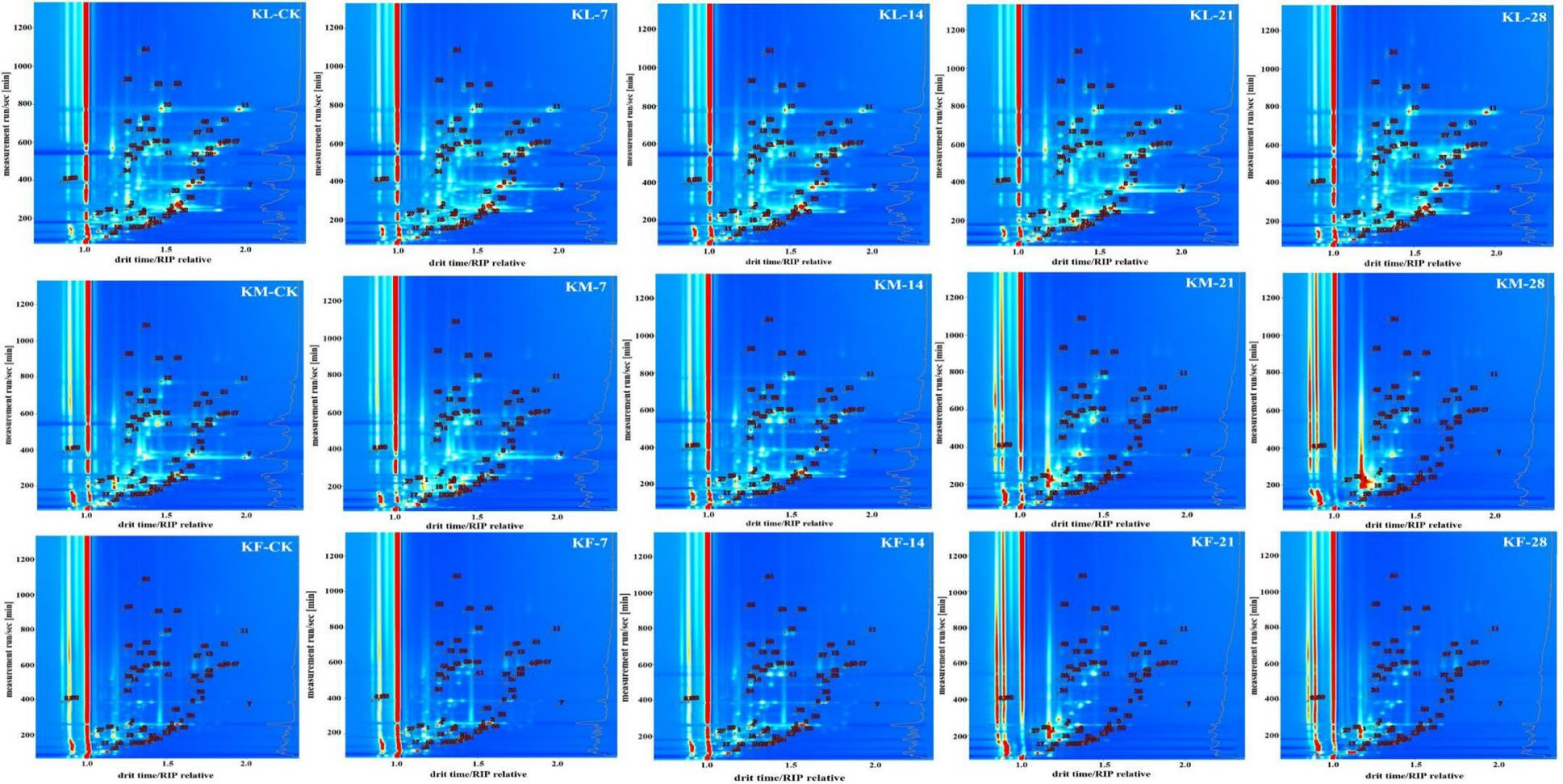
GC-IMS spectrum of the sample for qualitative analysis

To further analyze the changes in volatile compounds during storage in Kebab, qualitative analysis of substances can be performed via the database and IMS database. As shown in Fig. 4. The numbers in the figure indicate the characteristic peak position points of the volatile components of the samples. Each marked point represents a specific volatile component for qualitative analysis. The points on the entire spectrum represent all the volatile compounds detected in the sample [9]. There are 61 volatile substances that can be clearly qualitatively determined, including monomers and dimers of some volatile substances. The specific information is shown in Table 1.

As shown in Tables 1 and 61 volatile compounds were detected, including 13 aldehydes, 11 alcohols, 8 esters, 6 ketones, 1 acid, 2 furans, 1 ether, and 1 terpene. Aldehydes were the most abundant volatile components identified during the storage period of kebab, with a total of 13 volatile components. These include heptanal, butanal, 2-pentenal, 2-hexenal, and 5-methylfurfural. Second, the most abundant are alcohols, with a total of 11 kinds. These include 1-pentan-hexanol, 2-methyl-1-propanol, and 2-methylbutanol. Nonanal, hexanal, octanal, and heptanal are common volatile compounds found in meat products, primarily formed through the degradation of unsaturated fatty acids [27, 28]. Some alcohols may also be obtained by the reduction of aldehyde substances. Finally, the most abundant are esters, with a total of 8 kinds, such as ethyl acetate, ethyl2-methylpropanoate, butyl acetate, and hexyl acetate. Esters are important volatile compounds with relatively low thresholds but help improve the overall flavor quality of roasted mutton [9]. These ester and alcohol compounds, formed through slower esterification reactions and aldehyde oxidations [27]. There are a total of 6 types of ketones, such as 2-heptanone and 1-hydroxy-2-propanone. Ketones are intermediates in the formation of heterocyclic compounds and have relatively high flavor thresholds. The least common compounds are acids, furans, and terpenes. Wei et al. confirmed that hexal, octylaldehyde, 1-octene-3-ol, aldehyde, heptal, pentalaldehyde, 3-methylbutyraldehyde, and ethylfuran are key aroma compounds in roasted mutton [26].

**Table 1:** Determined compounds in GC-IMS (corresponding to the in Fig. 6)

Count	Compound	CAS#	Formula	MW	RI	Rt [sec]	Dt [RIPrel]
1	Trimethylamine	C75503	C3H9N	59.1	736.7	215.082	1.16806
2	Hexanal monomer	C66251	C6H12O	100.2	791	260.48	1.26303
3	Hexanal dimer	C66251	C6H12O	100.2	791.4	260.925	1.56777
4	1-pentanol monomer	C71410	C5H12O	88.1	774.6	245.345	1.25226
5	1-pentanol dimer	C71410	C5H12O	88.1	773.5	244.455	1.50929
6	Acetoin	C513860	C4H8O2	88.1	747.2	223.489	1.33909
7	n-Hexanol	C111273	C6H14O	102.2	878.4	356.72	1.99722
8	2-heptanone	C110430	C7H14O	114.2	893.3	373.866	1.6395
9	Heptanal	C111717	C7H14O	114.2	901.4	388.399	1.70066
10	n-Nonanal monomer	C124196	C9H18O	142.2	1108.9	776.21	1.46949
11	n-Nonanal dimer	C124196	C9H18O	142.2	1107.2	772.918	1.95338
12	1,8-Cineole monomer	C470826	C10H18O	154.3	1043.2	648.093	1.29598
13	1,8-Cineole dimer	C470826	C10H18O	154.3	1039.6	641.241	1.73041
14	2-heptenal (E) monomer	C18829555	C7H12O	112.2	961.3	494.61	1.26241
15	2-heptenal (E) dimer	C18829555	C7H12O	112.2	958.2	489.129	1.67261
16	1-hydroxy-2-propanone	C116096	C3H6O2	74.1	690.6	178.332	1.2327
17	2-Butanone monomer	C78933	C4H8O	72.1	593.3	135.077	1.07863
18	2-Butanone dimer	C78933	C4H8O	72.1	590.1	133.668	1.25106
19	Ethyl acetate	C141786	C4H8O2	88.1	606.2	140.713	1.3459
20	Acetone	C67641	C3H6O	58.1	524.4	105.018	1.13893
21	2-pentanone	C107879	C5H10O	86.1	688.8	176.879	1.37869
22	Butanal	C123728	C4H8O	72.1	595.4	136.017	1.3007
23	2-methyl-1-propanol	C78831	C4H10O	74.1	634.2	152.925	1.3758
24	3-methylbutanol	C590863	C5H10O	86.1	651.4	160.44	1.4148
25	Isopentyl alcohol	C123513	C5H12O	88.1	744.1	221.029	1.49135
26	Triethylamine	C121448	C6H15N	101.2	707.7	191.909	1.46391
27	2-ethylfuran monomer	C3208160	C6H8O	96.1	727.7	207.878	1.04794
28	2-ethylfuran dimer	C3208160	C6H8O	96.1	727.7	207.878	1.31514
29	2-pentenal (E)	C1576870	C5H8O	84.1	751.2	226.642	1.10843
30	Ethyl 2-methylpropanoate	C97621	C6H12O2	116.2	747.8	223.912	1.5742
31	2-methyl butanol	C137326	C5H12O	88.1	733.5	212.519	1.50588
32	2-Hexenal	C505577	C6H10O	98.1	848.9	324.331	1.52425
33	Butyl acetate	C123864	C6H12O2	116.2	818.5	290.833	1.61403
34	α-Pinene monomer	C80568	C10H16	136.2	925.3	430.71	1.22225
35	α-Pinene dimer	C80568	C10H16	136.2	922.7	426.068	1.67721
36	β-Pinene monomer	C127913	C10H16	136.2	972.3	514.26	1.22842
37	β-Pinene dimer	C127913	C10H16	136.2	972.9	515.292	1.64328
38	Beta-Pinene polymer	C127913	C10H16	136.2	973.5	516.323	1.72965
39	Octanal monomer	C124130	C8H16O	128.2	1011.4	586.297	1.40162
40	Octanal dimer	C124130	C8H16O	128.2	1009.9	583.329	1.83775
41	5-Methyl-furfural	C620020	C6H6O2	110.1	977.1	522.703	1.4787
42	1-octen-3-ol	C3391864	C8H16O	128.2	990.2	546.021	1.72872
43	Ethyl hexanoate monomer	C123660	C8H16O2	144.2	1005.6	574.85	1.33395
44	Ethyl hexanoate dimer	C123660	C8H16O2	144.2	1007.1	577.818	1.80767
45	2-Pentylfuran	C3777693	C9H14O	138.2	999.3	562.555	1.25687
46	2-Octanol	C123966	C8H18O	130.2	1010.4	584.177	1.4599
47	Hexyl acetate	C142927	C8H16O2	144.2	1010.6	584.601	1.89415
48	γ-Terpinene monomer	C99854	C10H16	136.2	1063.7	688.046	1.22492
49	γ-Terpinene dimer	C99854	C10H16	136.2	1063.4	687.481	1.70394
50	2-octenal (E) monomer	C2548870	C8H14O	126.2	1072.4	705.005	1.33771
51	2-octenal (E) dimer	C2548870	C8H14O	126.2	1068.5	697.495	1.82865
52	4-Terpineol	C562743	C10H18O	154.3	1177.7	910.284	1.22685
53	(E) -2-nonenal	C18829566	C9H16O	140.2	1165.1	885.757	1.41477
54	Cumin aldehyde	C122032	C10H12O	148.2	1259	1068.601	1.33543
55	1-nonanol	C143088	C9H20O	144.3	1165.7	886.827	1.53357
56	Myrcene	C123353	C10H16	136.2	990.9	547.161	1.29988
57	Limonene	C138863	C10H16	136.2	1034.2	630.577	1.65752
58	Butyl 2-methyl butanoate	C15706737	C9H18O2	158.2	1042.2	646.302	1.37294
59	2,3-butanedione	C431038	C4H6O2	86.1	587.6	132.59	1.16482
60	Ammonia monomer	C7664417	H3N	17	903.3	391.754	0.88942
61	Ammonia dimer	C7664417	H3N	17	901.3	388.096	0.84706

### PCA diagram of samples

**Fig. 5:**
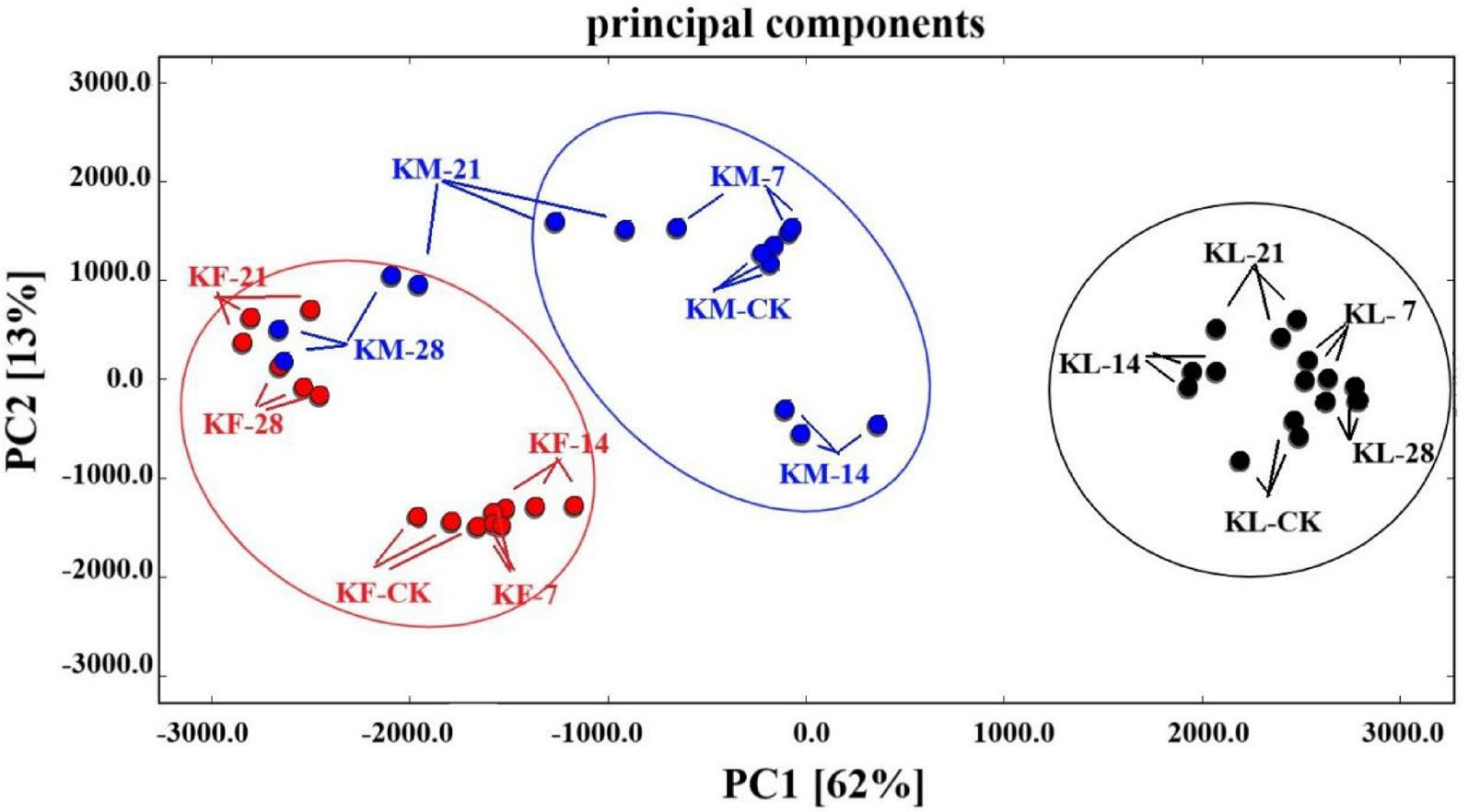
PCA diagram of samples

Consistent with the Gallery plot shown in Fig. 5, PCA can distinguish the five roasted mutton samples. If the samples are close to each other, there is a small difference. If they are far apart, the difference is large [29, 30]. The samples of KL, KF and KM within 28 days were subjected to cluster analysis via the PCA plug-in. The results are shown in Fig. 5. The results show that when GC-IMS and principal component analysis are used, the kebab at different refrigeration times can be identified. The variation contribution rates of PC1 and PC2 are 13% and 62%, respectively, and the cumulative variance contribution reaches 74%. For the first principal component (PC1), the distances between KF and KM are close, indicating that they have high similarity in volatile substance types and similar flavors. The distance on the 21st day was greater than that at the other times, indicating that there were obvious differences in the types and flavors of the volatile compounds. For the second principal component (PC2), the distances on the 7th, 14th, and 28th days of the KM samples are close, indicating that there is high similarity in volatile substance types and flavors and that the distance on the KM-21 samples is still greater than that at other times. Intuitively, the distance between the KL group and KM is very close, indicating that they have similar volatile compounds. This may contribute to the similarity in their volatile compound compositions.

### Sensory evaluation of the aroma

**Fig. 6:**
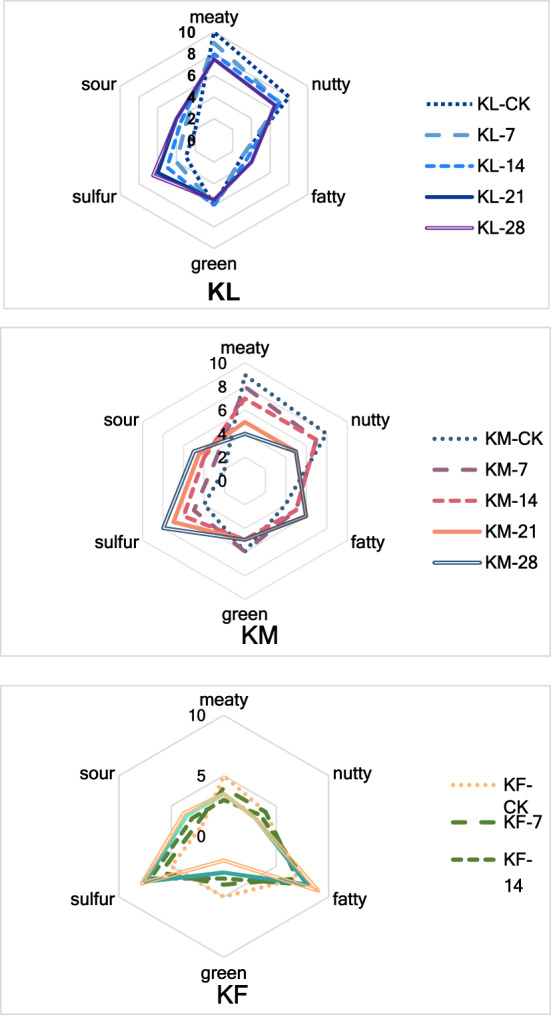
Aroma sensory evaluation results

The results of the sensory analysis of KL, KM and KF for Kebab stored for different durations are presented in Fig. 6. Sensory evaluation revealed that meat, nutty, fatty, green, sulfur, and sour aromas were the predominant aromas of the samples. In samples KL and KM, meat had the strongest aroma, and in sample KF, the fatty aroma was the most predominant. Among all the samples, the sour aroma was the weakest. When the storage time reached 28 days, sample KL maintained strong meaty and nutty aromas; in samples KM and KF, the fatty, sulfur, and sour aromas increased significantly. The results show that the sour and sulfur aromas in mutton became stronger when the storage time increased. While the fatty aroma became more evident in the KF when the storage time increased, its intensity was significantly weaker in the KL.

### Interaction between flavor substances that produce a sense of pleasure and serotonin (5-HT) receptors

#### Blood‒brain barrier permeability prediction

The blood‒brain barrier is a physiological barrier formed by vascular endothelial cells that limits the ability of many substances (such as most drugs) to enter brain tissue from the blood. Drugs that affect the central nervous system, including drugs that act on 5-HT receptors, usually need to be able to cross the blood‒brain barrier to have an effect. This study revealed that these key flavor substances can make people feel pleasure; therefore, predicting whether they have blood‒brain barrier penetration is a key indicator for determining their impact on the central nervous system. For this purpose, we used the deep learning-based multimodal model Deep-B3 [31] tool to predict six flavor compounds namely, 5-methylfurfural, 2-pentylfuran, (E)-2-hexenal, 2-octanone, trans-2-nonanal, and 3-methylbutanol. The results in Table 2 show that all the samples penetrated the blood‒brain barrier.

**Table 2:** The deep learning-based multimodal model Deep-B3

Name	mol_Name	Affinity (kcal/mol)	CAS	SMILES	Deep-B3 (A merged molecular representation deep learning-method for blood‒brain barrier permeability prediction)
mol1	5-methylfurfural	-44	620-02-0	CC1 = CC = C(01)C = 0	Permeable
mol2	2-pentylfuran	-55	3777-69-3	CCCCCC1 = CC=C01	Permeable
mol3	(E)-2-Hexenal	-44	6728-26-3	CCCC = CC=0	Permeable
mol4	2-Octanone	-48	111-13-7	CCCCCC(= O)C	Permeable
mol5	(E)-2-Nonenal	-52	18829-56-6	CCCCCCC = CC=0	Permeable
mol6	3-Methyl-1-butanol	-38	123-51-3	CC(C)CCO	Permeable

#### Molecular docking

**Fig. 7:**
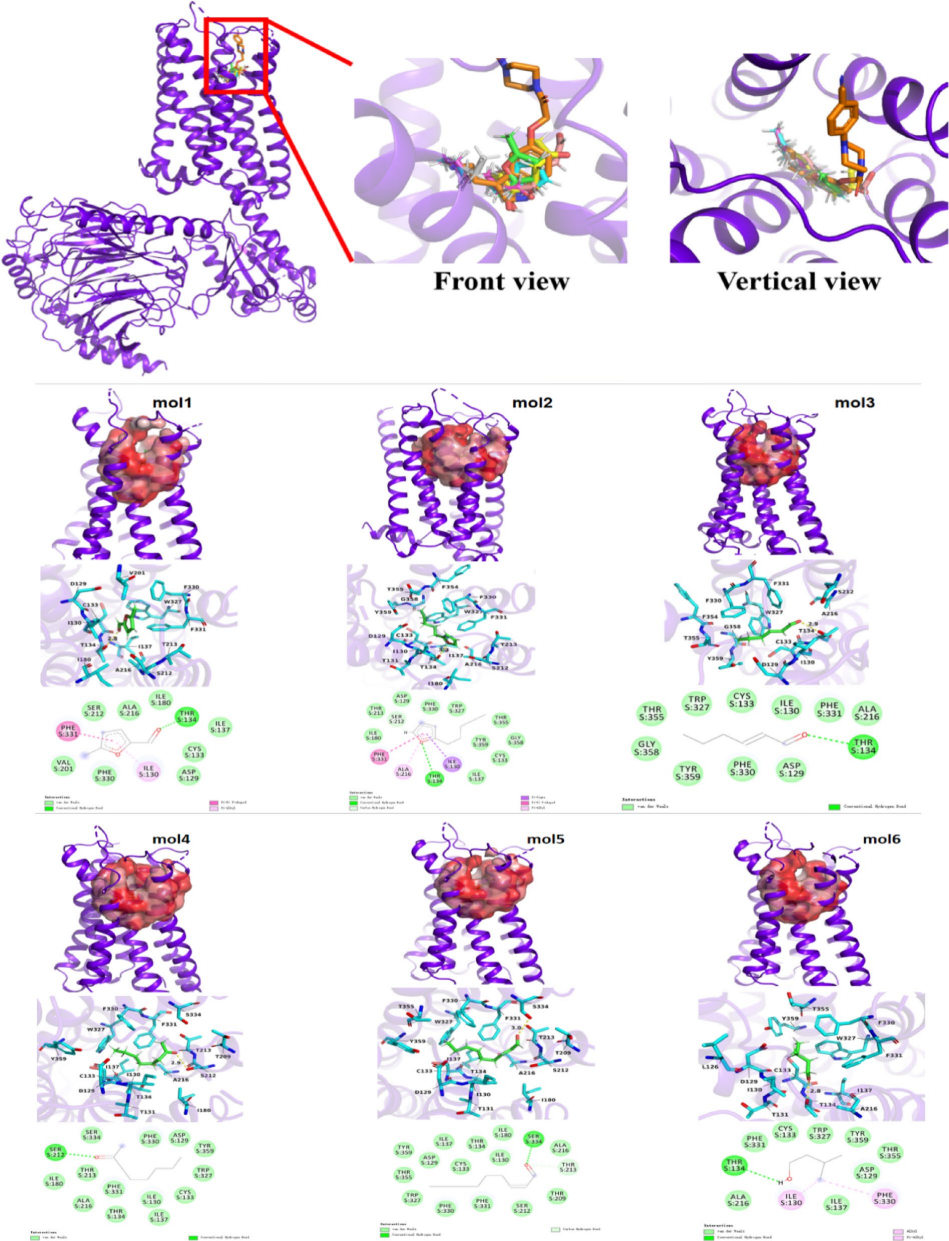
Comparison of the binding conformations of the six flavor molecules and the cocrystallized activator molecule

The best complex structure is selected according to the binding energy and binding conformation, and the Open Source PyMoL program is used for visual interaction analysis (Fig. 7).

The binding conformation of 5-methylfurfural to the serotonin receptor protein was determined by molecular docking, and the results are shown in Fig. 7. Shows the optimal complex conformation obtained by molecular docking, in which 5-methylfurfural binds to the highly hydrophobic pocket of the serotonin receptor protein, indicating that the two have a specific binding effect. Furthermore, the PyMoL and Discovery Studio 2016 client programs were used to map the interaction between 5-methylfurfural and the serotonin receptor protein. Panels show that 5-methylfurfural binds to serotonin receptors primarily through hydrophobic interactions, such as 331PHE\134THR\ILE130\. Importantly, these interacting residues are essentially consistent with serotonin and serotonin receptor binding, which may indicate that 5-methylfurfural exerts the same antidepressant effects as serotonin does.Molecular docking of 2-pentylfuran, (E)-2-hexenal, 2-octanone, trans-2-nonanal, and 3-methylbutanol was performed to obtain the binding conformation with the serotonin receptor, and the binding abilities were tested, as shown in Fig. 7. The optimal complex conformational map, binding capacity, and interactions of the serotonin receptor protein for each compound are shown. These key flavor substances can cross the blood-brain barrier, thereby inducing a sense of pleasure.

## Discussion

In this study, HS-GC-IMS technology was applied to characterize the fingerprint of volatile flavor substances in different tissues (lean meat, fat meat, and fat-lean mixed meat) of kebab during storage and combined with sensory evaluation. While the analysis of KL samples, The compounds with the highest concentrations in KL-7 mainly include 2-butanone, 5-methyl-2-furancarbaldehyde, E-2-pentanal, 2-pentylfuran, butyl acetate, butyl 2-methylbutyrate, 2-heptanone, 2,3-butanedione, etc. Up to 28 days of storage, the aroma compounds remained at a relatively high content and maintained strong meaty and nutty aromas. In comparison, the KF samples, the concentration of volatile compounds in KF-CK was low, and the concentrations in KF-7 and KF-14 showed no obvious changing trend. But in the KF-21 and KF-28 compounds changes to higher than those in the other samples, mainly trimethylamine, butyl 2-methylbutyrate, hydroxy-2-propanone, and ammonia, etc. The addition of KM samples, the concentration was the highest in KM-28, with 1-hydroxy-2-propanone, 2,3-butanedione, 2-methyl-1-propanol, trimethylamine, ammonia, etc., being the main substances. Besides, samples KM and KF, the fatty, sulfur, and sour aromas increased significantly. The results show that the sour and sulfur aromas in mutton became stronger when the storage time increased. Even at 28 days, the flavor was very similar to that of the KF sample. Compounds with unpleasant aromas are produced, resulting in off-flavors. Fingerprint spectra were used to analyze the volatile components in different storage periods to determine the differences in volatile components between samples, and the results were the same as those mentioned above. The KM and KF samples contained fat tissue, which eventually led to similarity. However, many substances detected by GC-IMS could not be qualified due to the limitations of the GC-IMS database, and these substances might play a crucial role in the flavor of grilled lamb, thus requiring further research in the future. Finally, the molecular mechanism of the production of a sense of pleasure by six key aroma compounds: 5-methylfurfural, 2-pentylfuran, (E)-2-hexenal, 2-octanone, trans-2-nonanal, and 3-methylbutanol, in kebab was verified via molecular docking simulation methods. The optimal complex conformational maps, binding capacities, and interactions of the serotonin receptor protein with each compound are presented. These key flavor substances can cross the blood-brain barrier, thereby inducing a sense of pleasure.

## Conclusions

At present, mutton production is mostly in the form of small workshops, with a small scale and inconsistent quality. kebab has soft and tender meat and a unique flavor. To achieve flavor fidelity, it is very important to study the characteristic qualities of traditional kebabs. During the storage process, the original quality of the lean meat tissue of roasted mutton is better than that of mixed meat and fatty meat. The aroma compounds of kebabs include mainly aldehydes, ketones, furans, alcohols and esters. Aroma deeply affects our emotions, memories and overall well-being. Eating kebabs helps to satisfy the hunger and produce a sense of pleasure is no exception with a healthy macronutrients content. Studies have shown that in addition to the impact of aroma on emotions, smell may also have physiological effects and help reduce feelings of anxiety and depression. Therefore, further research on the impact of aroma on emotions is highly important.

## Data Availability

The data that have been used are confidential.
